# Does Gender Predict Medical Students’ Stress in Mansoura, Egypt?

**DOI:** 10.3885/meo.2008.Res00273

**Published:** 2008-08-14

**Authors:** Mostafa Amr, Abdel Hady El Gilany, Aly El-Hawary

**Affiliations:** * Department of Psychiatry, Faculty of Medicine, Mansoura University, Egypt; † Department of Community Medicine, Faculty of Medicine, Mansoura University, Egypt; ‡ Department of Community Medicine, Faculty of Medicine, Zagazig University, Egypt

**Keywords:** gender, medical student, stress, depression, anxiety.

## Abstract

**Background:**

Medical education is perceived as being stressful with negative effects on students’ mental health. *However, few studies have addressed the influence of gender on stress in medical students.*

**Aim:**

To compare male and female medical students in Egypt on sources of stress, perception of stress, anxiety, depression, physical symptomatology, and personality profile.

**Methods:**

Data were collected through an anonymous self-administered questionnaire covering socio-demographic data, stressors, perceived stress scale, physical wellbeing factors, the Hospital Anxiety and Depression scale as well as neuroticism and extraversion subscales of the Eysenck Personality Questionnaire.

**Results:**

Stressors were reported by 94.5% of the total sample with equal gender proportions. Univariate analysis indicated that female students scored higher than males on depression and neuroticism scales while male and female medical students were similar on level of perceived stress, number of stressors, clinical anxiety, physical well-being factors and the extraversion scale. Multivariate logistic regression revealed that the independent predictors of a high stress level were the presence of more than five stressors, clinical anxiety and depression, and increased scores on the global sickness index and on the extraversion and neuroticism sub-scales.

**Conclusion:**

Despite there being no significant difference in perceived stress according to gender, females were less likely to cite relationship problems with teachers and substance abuse as sources of stress. Moreover, females scored significantly higher than males on depression and neuroticism scales.

Medical students experience substantial stress. Previous studies showed relatively high levels of distress among medical students such as symptoms of depression^1^ and suicidal thoughts.^2^ Despite increased attention being paid to research about the factors that contribute to the decline in students’ mental health,^3^ there are few studies that address the influence of gender on stress among medical students. Some studies suggest that female students have higher levels of stress than their male counterparts.^4^ Other studies do not reveal gender difference.^5^ There are also suggestions that gender may influence the way students in health care professions perceive stress.^6^

This study aims to explore the differences and correlates of perceived stress between female and male medical undergraduate students. We hypothesized that the female students in this college might report higher levels of perceived stress, anxiety and depression than their male counterparts.

## Materials and methods

A cross-sectional study of the six years of medical students of Mansoura College of Medicine in Egypt was conducted during February (after mid-year vacation) of the academic year of 2006/2007. After literature review, a preliminary questionnaire was designed in Arabic. This was pilot-tested on a sample of 32 students from different years over one-week period. The pilot group was not included in the actual study. An interview was also conducted after getting approval of the students. The questionnaire was then modified (e.g., rephrasing some questions and adding explanatory notes) accordingly in its final form. The questionnaire was approved by the college authority, as there is no formal research ethics committee.

Sample size was calculated using the Epi Info program (version 6.02). The total number of registered medical students in 2006 was 6808. From the pilot study, it is expected that around 23% of students suffer anxiety. With the worst acceptable level of 18%, the sample needed for the study was estimated to be at least 262 students for a power of 80% and confidence level of 95%. To overcome the sampling error of using the cluster-sampling technique, 10% was added for a total final sample size of 288 students.

Students were selected through a stratified cluster sampling technique. First, the students were stratified into the different academic years (first to sixth). From each year, a section or group (cluster) was randomly chosen. Students were allocated to these clusters for the practical teaching. All students in the chosen cluster were included for the study.

Participants completed an anonymous self-administered Arabic questionnaire covering socio-demographic factors, academic performance, sources of stress that might occur during the past twelve months, drug misuse, perceived stress scale (PSS), the Hospital Anxiety and Depression scale, assessment of physical well-being factors, and the neuroticism and extraversion subscales of the Eysenck Personality Questionnaire.

Sixteen potential sources of stress (stressors) were included. Students were asked to indicate the stressors, if any, affecting them. Perceived stress was measured by a previously validated 14-item perceived stress scale (PSS). The PSS has an internal consistency of 0.85 (Cronbach ∞ coefficient) and test-retest reliability during a short retest interval (several days) of 0.85.^7^ The Arabic version was tested among a sample of US Arab immigrants.^8^ The PSS does not tie appraisal to a particular situation; it is sensitive to the non-occurrence of events as well as to ongoing life circumstances. The stress score was stratified into no, mild, moderate (less than the first, second and third quartiles, respectively) (merged as low level) stress or severe (equal to or above the fourth quartile) (high level) stress.

The Hospital Anxiety and Depression Scale (HAD) is a brief, reliable self-report instrument that screens for the two most common aspects of neurosis (anxiety and depression). A score of eight or more for either the anxiety or the depression components denotes possible anxiety or depression.^9^ This cut off point has a sensitivity of 0.89, and specificity of 0.75.^10^ The Arabic version of the HAD scale was validated by El-Rufaie and Absood.^11^ The overall Cronbach ∞ measures of internal consistency were 0.78 and 0.87 for anxiety and depression, respectively.

A self–report questionnaire for assessing physical well-being factors, designed by Hojat et al., 12 included 15 health problems: a) somatic symptoms of stress including questions about skin rash, back pain, allergies, infectious diseases, frequent colds and generalized body pain; b) agitation symptoms e.g. sleep problems, headache, nausea, lack of appetite; c) eating/drinking and smoking problems; and d) chronic illness and health problems interfering with daily activities. The global sickness index was based on an average score obtained from all health problems listed in the questionnaire. The Cronbach ∞ coefficients for the four physical well-being factors were in the 0.90s.

For personality profile assessment, the Neuroticism and Extraversion subscales of the Arabic version of the Eysenck Personality Questionnaire were used. Test-retest reliability for neuroticism and extraversion scale scores of this instrument were 0.81 and 0.77 respectively, while the Cronbach ∞ coefficients of internal consistency was 0.76 and 0.81, respectively, among medical students.^13^ Neuroticism is a measure of emotional instability, while extraversion is a measure of sociability.

The investigators spent about 45 to 60 minutes in each class. Each class of student was briefed about the study objectives. The students gave fully informed verbal consent to participating. It was emphasized that all data collected were strictly confidential. Efforts were made to minimize underreporting, including asking staff to leave the classroom at the time of completing the questionnaire and strongly emphasizing to the students that the questionnaire was anonymous and would not be disclosed to their parents or staff.

Data were analyzed using SPSS version 11. For quantitative data, the unpaired student's t-test was used for group comparison. With categorical data, the chi-square test was used for comparisons between groups. Significant factors predicting stress on univariate analysis were entered into Wald multivariate logistic regression analysis. The outcome variable was the presence of a high level of stress as measured by the perceived stress scale. The predictor variables were all socio-demographic factors, the number of sources of stress, clinical anxiety, clinical depression, global sickness index, extraversion and neuroticism scores. P ≤ 0.05 was considered statistically significant in both univariate and multivariate analyses.

## Results

Of the 366 students asked to complete the study, 311 students did complete the questionnaire (85.0%). Of these, 164 (52.7%) were male and 147 (47.3%) female students. Thirty-nine students refused to participate and 16 eligible students were absent. The mean age of study participants was 20.7±2.4 years with a range of 16-25 years.

**Basic demographics** - Table 1 shows the socio-demographic characteristics of students. There were no significant differences in demographic factors between male and female students except the status of residence. Males were more likely to live outside campus than females.

**Table 1:** Socio-demographic data by gender of medical students

	Male students	Female students	Test statistic, p-value
Age:			
20	59(36)	40(27.2)	χ^2^=2.47, p = 0.098
20 and more	105(64)	107(77.8)	
X±SD	20.6±2.3	20.8±2.5	t = 0.67, p = 0.5
			
Number of stressors:			
Up to 5	137(83.5)	121(82.3)	χ^2^=0.8, p = 0.7
> 5	27(16.5)	26(17.7)	
			
Educational stage:			
Preclinical	103(62.8)	93(63.3)	χ^2^=0.007
Clinical	61 (37.2)	54(36.7)	p = 0.933
			
Academic performance of previous year:			
Excellent	44(26.8)	48(32.7)	
Very good	39(23.8)	44(29.9)	χ^2^=5.65
Good	41(25)	33(22.4)	p = 0.13
Pass	40(24.4)	22(15)	
			
Family residence:			
Urban	94(57.3)	91(61.9)	χ^2^=0.677
Rural	70 (42.7)	56(38.1)	p = 0.41
			
Student's resident during study:			
With family	128(78)	121(82.3)	χ^2^=7.81
University campus	14(8.5)	19(12.9)	p = 0.02
Outside campus	22(13.4)	7(4.8)	
			
Family income:			
Unsatisfactory	19(11.6)	16(10.9)	χ^2^=0.038
Satisfactory	145 (88.4)	131(89.1)	p = 0.85
Family size: Up to 5	97(59.1)	89(60.5)	χ^2^=0.063, p = 0.8
> 5	67 (40.9)	55(39.5)	
			
Father's education:			
Less than secondary	22(13.4)	25(17)	χ^2^=1.69,
Secondary	28(17.1)	30(20.4)	p = 0.43
Above secondary	114(69.5)	92(62.6)	
			
Father's work†:			
Professional/semiprofessional	119(72.6)	104(70.7)	χ^2^=0.3
Others	39 (23.8)	39(26.5)	p = 0.6
			
Mother's education†:			
Less than secondary	39(22.8)	33(22.4)	χ^2^=4.17
Secondary	30(18.3)	41(27.9)	p = 0.12
Above secondary	95(57.9)	73(49.7)	
Mother's work†: Housewives	77(47)	56(38.1)	χ^2^=2.17
Work outside home	85(51.8)	89(60.5)	p = 0.12

†Ten deceased fathers and four deceased mothers were excluded from analysis

**Sources of stress** -Table 2 shows that overall stressors were reported by 94.5% of male and female students. However, no gender difference was observed in the mean number of stressors. In univariate analysis, two stressors were found to be different between male and female students. Females were less likely to cite relationship problems with teachers (such as bad treatment, unfairness, over-criticism) and substance abuse (mostly minor tranquilizers) as stressors compared to male students.

**Table 2:** Events described as stressful† by gender of medical students

	No (%) reporting event		
	Male students	Female students	χ^2^ statistic, p-value
	(164)	(147)	
No stressful event	9(5.5)	8(5.4)	χ^2^=0.001, p = 0.9
X±SD	3.4±2.3	3.4±2.3	
Relationships problems with teachers	38(23.2)	15(10.2)	χ^2^=9.2, p = 0.002**
Academic problems	25(15.2)	30(20.4)	χ^2^=1.4, P = 0.2
Health problems (personal injury or illness)	36(22)	30(20.4)	χ^2^=0.11, p = 0.7
Death of a family member	10(6.1)	6(4.1)	χ^2^=0.65, p = 0.4
Emotional problems such as feeling of anxiety or	41(25)	50(84)	χ^2^=3.04, P = 0.08
depression			
Change of a family member's health	36(22)	31(21.1)	χ^2^=0.03, p = 0.85
Financial problems	36(22)	45(80.6)	χ^2^=3, p = 0.08
Relationships problems with opposite gender	34(20.7)	23(15.6)	χ^2^=1.3, p = 0.2
Relationships problems with parents or siblings	54(32.9)	54(36.7)	χ^2^=0.5, p = 0.5
Relationships problems with course-mates	42(25.6)	47(32)	χ^2^=1.5, p = 0.2
Accommodation problems	32(19.5)	36(24.5)	χ^2^=l.l, p = 0.3
Coping with the course of study	55(33.5)	50(34)	χ^2^=0.09, p = 0.9
Close contact with serious illness	18(11)	26(17.7)	χ^2^=2.9, p = 0.09
Substance abuse	45(27.4)	8(5.4)	χ^2^=26.5, p = 0.00**
Fear of failure in future career	24(14.6)	25(17)	χ^2^=0.3, p = 0.6
Others‡	27(16.5)	23(15.6)	χ^2^=0.04, p = 0.8

^†^Categories are not mutually exclusive

^‡^Part-time job, internet overuse, fear of death, peer competition

** Statistically significant p< 0.05

**Table 3:** Perceived stress, clinical anxiety and depression by gender of medical students (n, %)

	Male students	Female students	χ^2^statistic, p-value
Perceived stress:			
Mild/Moderate	136 (82.9)	112 (76.2)	χ^2^=2.18, p = 0.14
Severe	28 (17.1)	35 (23.8)	
			
Number of stressors:			
Up to 5	137 (83.5)	121 (82.3)	χ^2^=0.8, p = 0.7
> 5	27 (16.5)	26 (17.7)	
Clinical anxiety	31 (18.9)	35 (23.8)	χ^2^=1.1, p = 0.3
Clinical depression	35 (21.3)	53 (36.1)	χ^2^=8.3, p = 0.004

**Psychiatric morbidities -**Univariate analysis indicated that females had higher depression and neuroticism scores than their male counterparts, while both groups were similar on level of perceived stress, number of stressors, clinical anxiety, physical well being factors and the extraversion scale.

**Table 4:** Score of physical well-being factors and personality scale by gender of medical students

	Male students	Female students	Significance
	X±SD	X±SD	
Chronic health problems	3.5±1.5	3.5±1.6	t = 0.33, p = 0.74
Habits	4.4±1.7	4.7±1.7	t = 1.31, p = 0.19
Agitation symptoms	9.4±2.9	9.4±3.1	t = 0.17, p = 0.87
Somatic symptoms	11.5±3.4	11.8±3.5	t = 0.78, p = 0.44
Global sickness index	28.8±5.9	29.35±6.4	t = o.79, p = 0.43
Extraversion	14.6±4.5	14.8±4.8	t = 0.39, p = 0.69
Neuroticism	15.4±4.5	16.6±4.9	t = 2.4, p = 0.03

Globally, multivariate logistic regression analysis revealed that the independent predictors of high stress level among the total sample were presence of more than five sources of stress, presence of clinical anxiety or depression, a high score on the global sickness index and high scores on the extraversion and neuroticism scales (Table 5).

**Table 5:** Multivariate logistic regression analysis of significant predictors of severe stress among studied students.

			High stress level
	β	p	Odds Ratio (95% confidence interval)
Predictor			
Number of stressors:			
Less than 5	−		1 (r=reference group)
5 or more	1.1	0.004	2.95(1.4–6.2)
Anxiety: No	−		1 (r)
Yes	1.1	0.004	2.9(1.4–6.1)
Depression: No	−		1 (r)
Yes	0.7	0.02	2.1 (1.1–4.0)
Global sickness index(continuous)	0.01	0.000	1.1(1.05–1.2)
Extraversion (continuous)	0.07	0.03	1.1(1.01–1.2)
Neuroticism (continuous)	0.08	0.04	1.1(1.003–1.2)
Constant	−7.8		
Percent correctly predicated	83.9%		
Model χ^2^	69.6, p = 0.000		

## Discussion

In the present study, female students reported a slightly higher level of perceived stress (23.8%) compared to males (17.1%). However, this difference was not statistically significant on both univariate and multivariate analysis. In addition, gender was not a significant factor in stress reporting, as mean number of stressors was similar in both male and female students. These findings are similar to those of other studies.^14^,^15^ However, Dahlin et al.^1^ suggested greater stress among female students.

The lack of a gender difference in our study may reflect the contemporary changes in medical schools in Arab countries.^15^ There are more female students entering medical schools. Mule and Barthel^16^ described the social changes in Egypt, where there has been an increase in women's participation in the work force and, to some extent, in political life. Furthermore, globalization and exposure to Western culture have steered this traditionally Islamic country with alternative gender ideologies. In addition, lack of difference between the genders may be due to the highly selective and homogenous nature of the student population with unique personal characteristics desirable for the competitive environment of medical school.^17^

Two stressors were significantly associated with gender, with female students less likely to report relationship problems with teachers and substance abuse as stressor. However, it is not clear from this study why the females were less likely to have problems with teachers.^18^ There are several possible explanations. Female students have been found to out-perform the males, especially in clinical subjects.^19^ Recently there is a trend towards increased recruitment of female staff in this medical school (accounting for about 32% of staff) with a possibility of better communication between the same gender. Finally, male staff might have more empathic attitude towards women students in an Islamic conservative society.

Consistent with a recent study of major stressors for students, 20 the importance of substance abuse is also evident in this study, with males more likely than females to cite it as a stressor. This is consistent with the findings of a nationwide study conducted among the Egyptian general population.^21^ Low drug abuse rates among women in Egypt may be attributed to the values of a traditional society that established sanctions against women's drug abuse and kept them on a higher moral pedestal. 23 This contrasts to studies from Western countries, where female college students have almost the same level of alcohol and illicit drug use.^22^

In the present study, women scored higher on a depression scale in the clinically significant range. This result is similar to findings of Dahlin et al.^1^ and Ahmadi et al.^24^ in Swedish and Iranian medical students, respectively. There are also conflicting results indicating no gender difference.^2^ In a study among doctors, Tyssen et al^15^ found a higher prevalence of mental problems among male doctors than the general male population; whereas findings about female doctors did not differ from the generally high prevalence rates of mental problems among the general female population. In developing countries, women are more likely to experience depression than men.^26^ Other data from less developed countries are inconsistent, sometimes finding no gender difference in depression.^27^ The most likely explanation of gender differences is multifactorial, including biological, sociocultural, or variable combinations of each.

Few studies have focused on gender comparisons on the personality of medical students.^28^ In the present study, females scored higher than males on a neuroticism scale. The neuroticism dimension represents emotional instability. Individuals who score high on this dimension tend to have emotional problems such as anxiety, depression, and low self-esteem.^29^ They tend to be more sensitive and more self-doubting.^30^

In conclusion, this study among the medical students of a particular institute in Egypt shows that there is no significant difference of perceived stress between the male and female students. Female students are less likely to cite relationship problems with teachers and substance abuse but scored significantly higher than males on depression and neuroticism scales. Female students are similar to males regarding perceived stress, number of stressors, anxiety symptoms, and a global sickness index.

The generalizability of the study result is limited by the sample being recruited from a single medical school and a lack of baseline information concerning the mental health of medical students in Egypt. Furthermore, there is a lack of population-based data to support our results. As more and more women physicians enter into the professional work force in our country, with their psychosocial characteristics revealed as students, future research studies on possible links between gender and outcome measures such as medical practice and doctor-patient relationships are demanded.
